# Effectiveness of a multi-loop traction device for colorectal endoscopic submucosal dissection performed by trainees: a pilot study

**DOI:** 10.1038/s41598-022-14407-3

**Published:** 2022-06-17

**Authors:** Ryuhei Jinushi, Tomoaki Tashima, Rie Terada, Kazuya Miyaguchi, Hiromune Katsuda, Tomoya Ogawa, Yuya Nakano, Yoichi Saito, Akashi Fujita, Yuki Tanisaka, Masafumi Mizuide, Yumi Mashimo, Tomonori Kawasaki, Shomei Ryozawa

**Affiliations:** 1 Department of Gastroenterology, Saitama Medical University International Medical Center, 1397-1 Yamane, Hidaka, Saitama 350-1298 Japan; 2 Department of Pathology, Saitama Medical University International Medical Center, Hidaka, Saitama Japan

**Keywords:** Colonoscopy, Gastrointestinal diseases, Materials for devices

## Abstract

Colorectal endoscopic submucosal dissection (ESD) is a difficult procedure, and its introduction to trainees has been debated. Although the criteria for performing colorectal ESD vary among institutions, it is often allowed after gaining experience performing surgeries in animals and upper gastrointestinal ESD. This pilot study aimed to compare the treatment outcomes of ESD performed by trainees using the multi-loop traction device (MLTD group) and those of conventional ESD performed by experts (control group). It also aimed to determine whether the MLTD can be used to safely introduce colorectal ESD to trainees. We included 26 colorectal ESD patients (13 in the MLTD group and 13 in the control group) treated at our hospital from October to December 2021. There were no significant differences in the procedure time (50 min vs. 30 min), dissection speed (19.9 mm^2^/min vs. 28.7 mm^2^/min), and intraoperative perforation (0% vs. 0%) of the two groups. Furthermore, the rate of ESD self-completion in the MLTD group was 100%. Therefore, the use of the MLTD allowed the safe introduction of colorectal ESD, even among endoscopists with no experience performing colorectal ESD. Consequently, the use of the MLTD may replace animal and upper gastrointestinal ESD when introducing colorectal ESD to trainees.

## Introduction

Colorectal cancer (CRC) is a common fatal disease. Approximately 150,000 new cases of colorectal cancer are diagnosed annually, and approximately 50,000 people in the United States are expected to die from colorectal cancer each year^1^. Recently, the importance of endoscopic submucosal dissection (ESD) for early-stage colorectal cancer has increased; however, colorectal ESD remains a challenging procedure. Colorectal ESD has a high rate of intraoperative perforation (2.6%) compared to other procedures, thus rendering its introduction to trainees difficult^2^. The appropriate introduction of and training for colorectal ESD for trainees is still undergoing discussion^3^. Additionally, the criteria for performing colorectal ESD vary among institutions and have not yet been established; however, the performance colorectal ESD is often allowed after gaining experience performing animal and upper gastrointestinal ESD^4^. Recently, the use of a traction device for gastrointestinal ESD has been applied to colorectal ESD because it improves the visual field of the submucosa, thereby shortening the procedure time, increasing the dissection speed, and decreasing intraoperative perforations^5,6^. Although there have been reports of colorectal ESD using traction devices for those who have gained a certain level of experience performing ESD in animals and the upper gastrointestinal tract^5^, to the best of our knowledge, there have been no reports of colorectal ESD performed using traction devices by those who have not been trained to perform colorectal ESD. This study compared the treatment outcomes of ESD performed by the trainee group using a multi-loop traction device (MLTD; Boston Scientific Co. Ltd., Tokyo, Japan) with those of ESD performed by experts using the conventional ESD method.

## Results

### Patient characteristics

We included 26 patients in this analysis (MLTD group, 13 patients; control group, 13 patients). There were no significant differences in age, tumor location, horizontal location, morphology, estimated tumor size, history of abdominal surgery, preoperative biopsy, and antithrombotic therapy between groups (Table 1). A total of 11 preoperative biopsy procedures were performed in both groups; these were performed at a previous hospital before the patients were referred to our department.

**Table 1:** Patient characteristics.

	MLTD group (n = 13)	Control group (n = 13)	P value
**Age, mean (SD)**	73.2 (10.7)	66.9 (7.4)	0.1
**Tumor location, n (%)**			0.33
Right colon	8 (61.5)	6 (46)	
Left colon	4 (30.8)	3 (23)	
Rectum	1 (7.7)	4 (31)	
**Horizontal location, n (%)**			0.68
One-quarter	9 (69.2)	8 (61.5)	
One-quarter to one-half	4 (30.8)	5 (38.5)	
**Morphology, n (%)**			0.11
LST-G	3 (23.1)	6 (46)	
LST-NG	10 (76.9)	5 (39)	
Others^a^	0 (0)	2 (15)	
**Estimated tumor size, mm (IQR)**	20 (20–30)	25 (20–30)	0.57
**Surgical history, n (%)**	4 (30.8)	3 (23.1)	0.66
**Preoperative biopsy, n (%)**			0.23
Yes	4 (30.1)	7 (53.9)	
**Antithrombotic therapy, n (%)**			1.0
Yes	1 (7.7)	1 (7.7)	

*SD* standard deviation, *IQR* interquartile range, *LST-G* laterally spreading tumor granular type, *LST-NG* laterally spreading tumor non-granular type.

^a^Refers to 0-Is lesions that could not be classified as laterally spreading tumors.

### Treatment outcomes

The rate of ESD self-completion in the MLTD group was 100%. There were no statistically significant differences in the procedure time (50 min vs. 30 min; p = 0.38), dissection speed (19.9 mm^2^/min vs. 28.7 mm^2^/min; p = 0.19), and intraoperative perforation (0% vs. 0%; p = 1.0) between groups. Additionally, there was no significant difference in adjunctive outcomes (Table 2). Regarding the procedure time, the 20-min difference between groups was clinically acceptable considering the high curability and low complication rates.

**Table 2:** Treatment outcomes.

	MLTD group (n = 13)	Control group (n = 13)	P value
**MDZ dose, mg (IQR)**	3 (3–4)	2.5 (2–4)	0.62
**MucoUp dose, mL (IQR)**	28 (15–40)	20 (16–40)	0.64
**Maneuverability, n (%)**			0.4
Good/normal	10 (76.9)	8 (61.5)	
Poor	3 (23.1)	5 (38.5)	
**Poor submucosal lifting because of fibrosis, n (%)**			1.0
Positive	0 (0)	0 (0)	
Procedure time, min (IQR)	50 (33–63)	30 (23–55)	0.38
Dissection speed, mm^2^/min (IQR)	19.9 (14.4–26)	28.7 (17–35.7)	0.19
**Specimen damage, n (%)**			1.0
Yes	0 (0)	1 (7.7)	
**En bloc resection, n (%)**			1.0
Yes	100 (13)	100 (13)	
**Resectabillity, n (%)**			1.0
R0	100 (13)	100 (13)	
**Resected tumor size, mm (IQR)**	30 (22–33)	24 (20–36)	0.92
**Largest diameter of the resected specimen, mm (IQR)**	43 (32–45)	34 (30–45)	0.74
**Smallest diameter of the resected specimen, mm (IQR)**	30 (22–33)	33 (24–38)	0.61
**Final pathology, n (%)**			
Histology			0.12
Adenoma	9 (69.2)	5 (38.5)	
Tub	4 (30.8)	8 (61.5)	
Por, sig, muc	0 (0)	0 (0)	
Depth			0.29
Tis	3 (23.1)	6 (46.2)	
T1a	1 (7.7)	2 (15.4)	
T1b	0 (0)	0 (0)	
**Intraoperative perforation, n (%)**			1.0
Yes	0 (0)	0 (0)	
**Intraoperative bleeding, n (%)**			1.0
Yes	0 (0)	0 (0)	
**Delayed perforation, n (%)**			1.0
Yes	0 (0)	0 (0)	
**Delayed bleeding, n (%)**			1.0
Yes	0 (0)	0 (0)	
**Surgery due to adverse events, n (%)**			1.0
Yes	0 (0)	0 (0)	
**Recurrence, n (%)**			1.0
Yes	0 (0)	0 (0)	

*IQR* interquartile range, *MZD* midazolam.

Three patients who required additional traction using the intermediate loop of the MLTD and no patients who required a new MLTD were added to the MLTD group to obtain more information about MLTD. Additionally, specimen damage and muscle layer damage associated with traction were not observed (Table 3).

**Table 3:** Treatment outcomes of the MLTD group.

**Additional traction with the MLTD intermediate loop, n (%)**	3 (23.1)
**New MLTDs added, n (%)**	0 (0)
**MLTD dropouts, n (%)**	0 (0)
**Timing of MLTD installation, n (%)**	
Before the full circumferential mucosal incision	4 (30.8)
After the full circumferential mucosal incision	9 (69.2)
**Damage to the specimen after traction, n (%)**	0 (0)
**Damage to the muscle layer after traction, n (%)**	0 (0)

*MLTD* multi-loop traction device.

## Discussion

Although colorectal ESD results in superior treatment outcomes compared to those of endoscopic mucosal resection (EMR), such as higher en bloc resection rates, it is difficult to standardize the training system because of poor maneuverability and the risk of intraoperative perforation^7^. To date, the introduction of and training methods for colorectal ESD for trainees are still being discussed, and workshops and hands-on seminars are being actively conducted^3,4^. On average, the amount of endoscopic experience of the operator at the time of human ESD initiation is 7.7 years (standard deviation [SD], 4.1), and it is generally initiated in the gastric antrum (90%)^4^. Recently, the use of traction devices for gastrointestinal ESD has been reported to shorten the treatment time, increase the speed of submucosal dissection, and reduce intraoperative complications^5,6^. Traction devices also have been applied to colorectal ESD, but the threshold for their introduction to trainees remains high. A literature search of “Multi-loop traction device” in PubMed from 2000 onward revealed six procedures^6,8–12^, and only two of those were relevant to this study^6,8^. To the best of our knowledge, no report pertaining to trainees with no experience performing colorectal ESD exist in the literature. We compared the treatment outcomes of the MLTD group who underwent ESD with the MLTD performed by trainees and those of the control group who underwent ESD without the MLTD performed by experts and found no significant differences in primary and secondary outcomes. The use of MLTD enabled the safe introduction of colorectal ESD to trainees without experience performing ESD in animals or upper gastrointestinal tract ESD. This study had some limitations. First, it was performed at a single institution. Second, it involved a small number of patients. Because of the rate of intraoperative perforation associated with colorectal ESD (2.6%)^2^ and the small number of patients in this study, we chose procedure time rather than intraoperative perforation as the primary study outcome. If there had been a significant difference in the procedure time between groups, then the introduction of MLTD for beginners might be questionable. However, we did not observe a clear significant difference between groups; therefore, we will continue to accumulate more data for further investigation. Of course, we believe that there will be significant differences in the procedure time, the speed of submucosal dissection, and intraoperative complications when using traction devices for gastrointestinal ESD among endoscopists of the same level. Therefore, we conducted this study to examine what considerations are necessary so that experts can perform colorectal ESD as safely as they perform conventional ESD when introducing it to beginners (trainees performing ESD with the MLTD vs. experts performing ESD with the conventional method). In the future, the outcomes of a large number of patients should be compared between these two groups using the propensity score matching method to reduce selection bias, and this study should be linked with a randomized controlled trial.

## Methods

### Study design and ethical statements

This was a retrospective cohort study conducted at the Saitama Medical University International Medical Center in Japan. It was approved by the Institutional Review Board of Saitama Medical University International Medical Center (institutional ID: 20-202) and performed in accordance with the principles of the Declaration of Helsinki. We obtained informed consent from all patients before their inclusion in this study.

### Patients

We included 26 consecutive patients who underwent colorectal ESD at our hospital between October and December 2021. All procedures were conducted according to the 2019 Japanese Society for Cancer of the Colon and Rectum (JSCCR) guidelines for the treatment of colorectal cancer^13^ for patients with a preoperative endoscopic or pathologic diagnosis of early colorectal cancer.

### Trainees and experts

Two trainees and three experts participated in the study. The trainees had experience performing fewer than ten upper gastrointestinal ESD procedures and had no experience performing colorectal ESD. The experts had experience performing more than 200 gastrointestinal ESD procedures. Both the trainees and experts were physicians at our hospital.

### Definitions of the MLTD group and the control group

The MLTD group included patients who underwent colorectal ESD with the MLTD performed by the two trainees. The control group included patients who underwent colorectal ESD without the MLTD performed by the three experts.

### Colorectal ESD setting

We performed colorectal ESD in the endoscopy room under intravenous anesthesia using midazolam and pethidine. Scopolamine was used as an antispasmodic drug; however, glucagon was substituted in patients with cardiac disease or benign prostatic hyperplasia. All ESD procedures were performed using therapeutic endoscopes (PCF-H290ZI or GIF-H290T; Olympus, Medical Systems Co., Tokyo, Japan) with a transparent cap (Olympus). Generally, PCF-H290ZI is used for colorectal ESD and GIF-H290T is often used for rectal lesions. We used a 1.5-mm DualKnife J (KD655Q; Olympus) to perform the mucosal incision or submucosal dissection. Using an electrosurgical generator (VIO 3; ERBE Elektromedizin, Tübingen, Germany), the endoCUT I mode (effect 1 or 2, duration 2, interval 2) was used for the mucosal incision, forced coagulation (effect 4.5 or 6.1) was used for the submucosal dissection, and spray coagulation (effect 1.2) was used for hemostasis. To elevate the submucosa, a local injection of 0.4% sodium hyaluronate (MucoUp^®^; Boston Scientific, Tokyo, Japan) and a small amount of indigo carmine were administered.

### Strategies for performing colorectal ESD using the MLTD

**Figure 1:**
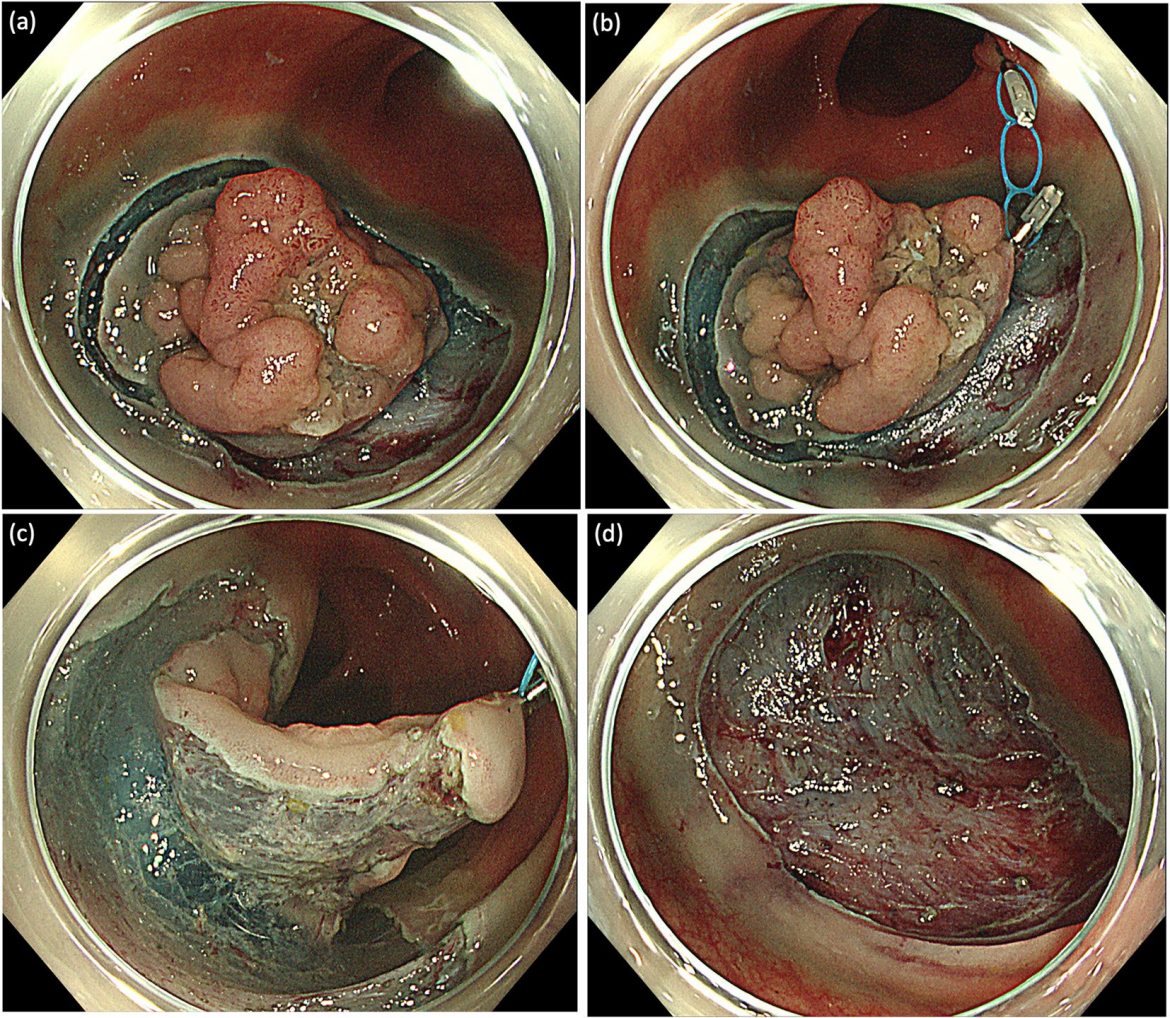
Strategies used for colorectal endoscopic submucosal dissection (ESD) using the multi-loop traction device (MLTD). (**a**) Full circumferential mucosal incision around the lesion. (**b**) Attaching the MLTD on the mucosal edge to elevate it. (**c**) The visual field of the submucosa and muscle layer is good, allowing for safe submucosal dissection. (**d**) En bloc resection of the lesion was successful and without intraoperative complications.

Step 1: Before or after the full circumferential mucosal incision was performed around the lesion (Fig. 1a), we attached the MLTD to the mucosal edge for elevation (Fig. 1b). The MLTD was attached to either the lesion or the normal mucosa, contralateral to the lesion. As a precaution, sufficient MucoUp^®^ was injected in the submucosa at the site of the MLTD attachment to avoid traction on the muscle layer.

Step 2: The direction, distance, and gravity should be checked thoroughly before attaching the MLTD because the traction effect depends on the MLTD attachment site.

**Figure 2:**
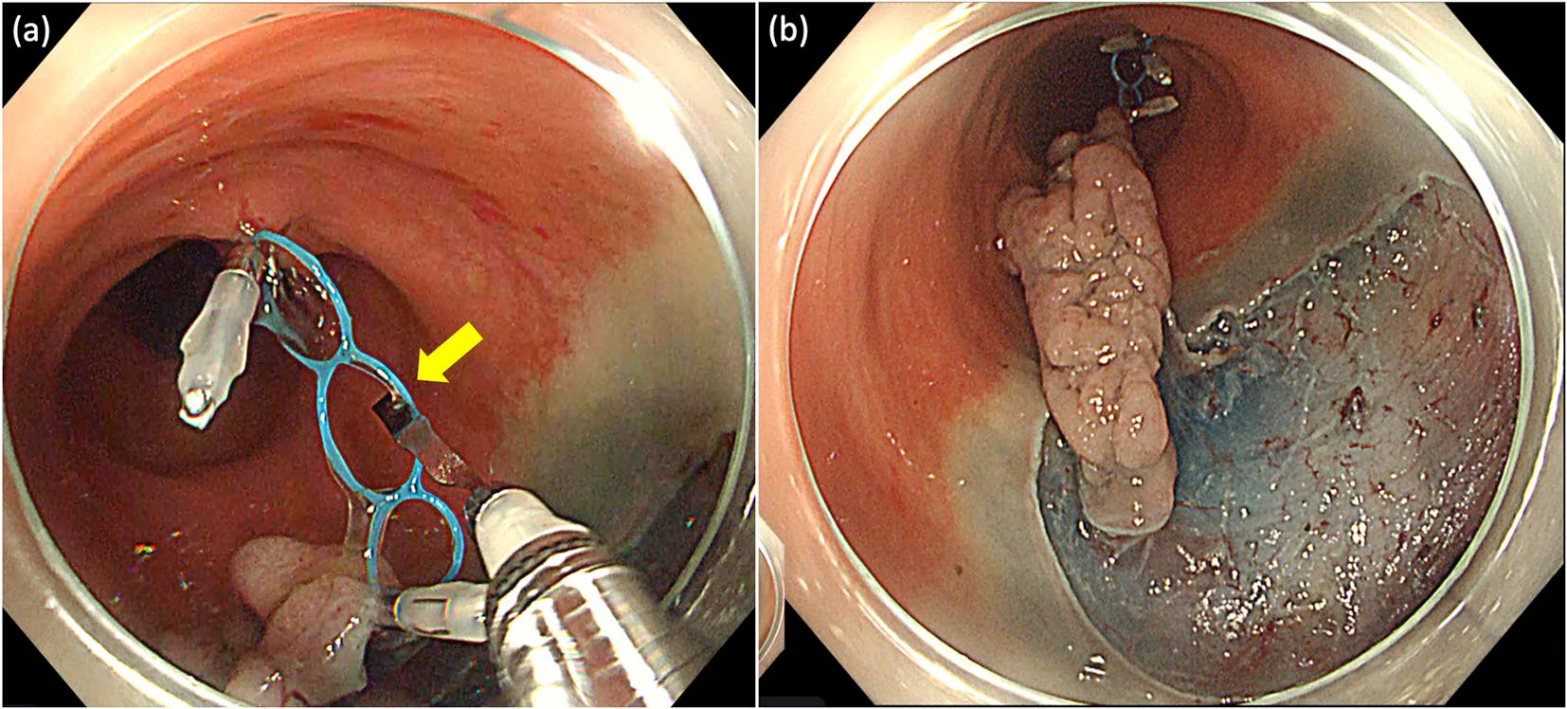
Additional traction. (**a**) When the traction effect was inadequate, additional traction was applied using the hole in the middle (yellow arrow). (**b**) After additional traction.

Step 3: After attaching the MLTD, a good visual field of the submucosa was obtained, and the submucosa was dissected safely and easily (Fig. 1c,d). When the traction effect was inadequate, additional traction was provided using the hole in the middle (Fig. 2a,b) or a new MLTD was prepared.

Step 4: After lesion resection, we removed the MLTD with a biopsy forceps and collected the lesion.

### Advantages of the MLTD

There are several traction methods for colorectal ESD, including the use of the S–O clip^14^, ring-shaped thread traction^15^, and the cross-counter technique^16^. If the S–O clip or ring-shaped thread traction is used, then the traction may weaken as the submucosal dissection progresses, and additional devices may be required. With the cross-counter technique, the traction force can be adjusted to some extent, but a balloon-type overtube is required. Compared to these traction methods, the MLTD has the following five advantages: it is easy to prepare; it can be attached with ordinary rotary clip devices without the need for additional specialized devices; it allows the ability to deliver through the scope to the surgical field; additional traction is possible using the intermediate loop and the traction direction can be adjusted; and it is easy to cut it with biopsy forceps.

### Definitions

During this study, procedure time was defined as the time from the initial mucosal incision to the tumor resection. Dissection speed was calculated using the following equation: largest diameter of the resected specimen (mm)/2 × smallest diameter of the resected specimen (mm)/2 × 3.14/procedure time (minutes). Intraoperative perforation was defined as perforation during ESD. The midazolam dose was defined as the total amount of midazolam used during ESD. The MucoUp^®^ dose was defined as the total amount of MucoUp^®^ used during ESD. Specimen damage was defined as any damage to the specimen during ESD, including that caused by traction. Endoscopic en bloc resection was defined as resection of one piece that included the whole tumor. Tumor resectability was evaluated based on its final pathology. Intraoperative bleeding was defined as bleeding that could not be treated by endoscopic hemostasis during ESD. Delayed perforation was defined as a perforation diagnosed by computed tomography after ESD. Delayed bleeding was defined as hemorrhaging that required endoscopic hemostasis after ESD. Cases that required surgery because of ESD-related complications were defined as surgery due to adverse events. Regarding maneuverability, three expert endoscopists with experience performing more than 200 gastrointestinal ESD procedures divided the cases into two groups (good/normal or poor) based on the tumor location and intestinal peristalsis.

### Statistical analysis

We compared the treatment outcomes of the MLTD group (two trainees) and those of the control group (three experts). The primary study outcome was the procedure time. The secondary outcomes were dissection speed and intraoperative perforation. Adjunctive outcomes were the midazolam dose, MucoUp^®^ dose, maneuverability, specimen damage, en bloc resection, resectability, resected tumor size, largest diameter of the resected specimen, smallest diameter of the resected specimen, final pathology, intraoperative bleeding, delayed perforation, delayed bleeding, surgery due to adverse events, and recurrence. In the two groups, binary variables were evaluated using Pearson’s chi-square test, and continuous variables were evaluated using the Mann–Whitney U test or Student’s t-test. All analyses were performed using STATA^®^ version 17 (StataCorp, College Station, TX, USA), and statistical significance was set at p < 0.05.

### Informed patient consent statement

Informed consent was obtained from all patients who participated in this study.

## Data Availability

The data that support the findings of this study are available from the corresponding authors (TT or RJ) upon reasonable request.

## Abbreviations

MLTD: Multi-loop traction device
EMR: Endoscopic mucosal resection
ESD: Endoscopic submucosal dissection
CRC: Colorectal cancer
LST-G: Laterally spreading tumor granular type
LST-NG: Laterally spreading tumor non-granular type
JSCCR: Japanese Society for Cancer of the Colon and Rectum
MDZ: Midazolam
R0: No tumor identified at the lateral or vertical margins
SD: Standard deviation
IQR: Interquartile range
